# 335. Factors Associated with Loss to Follow-Up in Outpatient Parenteral Antimicrobial Therapy: A Retrospective Cohort Study

**DOI:** 10.1093/ofid/ofad500.406

**Published:** 2023-11-27

**Authors:** Christina M Kaul, Matthew Haller, jenny Yang, Sadie Solomon, Maria Khan, Robert Pitts, Michael Phillips

**Affiliations:** NYU Grossman School of Medicine, New York, New York; NYU Grossman School of Medicine, New York, New York; New york university, new york city, New York; NYU Langone Health, New York, New York; NYU Grossman School of Medicine, New York, New York; NYU Langone Health, New York, New York; New York University, New York, NY

## Abstract

**Background:**

Outpatient parenteral antimicrobial therapy (OPAT) has long been utilized to treat infectious conditions in the outpatient setting, particularly for those conditions requiring prolonged courses of antimicrobials. It is well-known that follow-up with Infectious Diseases can reduce hospital readmission. However, factors associated with loss to follow-up are not well-studied. We sought to understand factors associated with an increased risk to loss of follow-up with Infectious Diseases in OPAT patients.

**Methods:**

This was a retrospective cohort study conducted at NYU Langone Health (NYULH). The study was approved and granted a waiver of authorization and informed consent by the NYU IRB. All OPAT episodes for patients at least 18 years of age initiated during admission from January 1, 2017 to December 31, 2020 were screened.

Data were analyzed using SPSS v.28 and Microsoft Office Excel 2019. Descriptive statistics were used. A multivariate logistic regression model was used to examine factors associated with risk of loss to follow-up.

**Results:**

1,846 instances qualifying OPAT courses were identified. 1,528 (82.7%) of patients were recommended for follow-up with ID. 1,110 (72.6%) patients were seen, while 418 patients (27.4%) were not. Of the patients seen by ID, 981 (88.4%) were seen by the physician, and 129 (11.6%) were only seen by the NP. Patients were seen or contacted by the ID physician a median of two times during their follow-up period (IQR 1-3). The median follow-up period for those patients not lost to follow-up was 21 days (IQR 10-36).

The model was adjusted for chronic medical condition, hospital of admission, and follow-up duration. There was a significant association noted between loss to follow-up with ID and discharge to SAR (OR 3.242, p < 0.001) or LTC (OR 5.906, p < 0.001). There was also an association between the use of federal insurance and loss to follow-up (OR 1.493, p = 0.029).

**Conclusion:**

Discharge to SAR and LTC are strongly associated with loss to follow-up with Infectious Diseases. This work underscores the importance of maintaining links to patients discharged to sub-acute healthcare facilities. Transitions of care remain a weakness in healthcare delivery, and must be improved upon in OPAT.

**Disclosures:**

**All Authors**: No reported disclosures

